# Infectious keratoconjunctivitis in European bison (*Bison bonasus*) in Poland: risk factors, epidemiology and anatomopathological changes with analysis of potential role of *Thelazia* nematodes in the disease development

**DOI:** 10.1186/s12917-024-04375-3

**Published:** 2024-11-21

**Authors:** Katarzyna Filip-Hutsch, Aleksander W. Demiaszkiewicz, Tomasz Hutsch, Karolina Duk, Daniel Klich, Anna M. Pyziel, Paulina Balińska, Krzysztof Anusz

**Affiliations:** 1 Department of Food Hygiene and Public Health Protection, Institute of Veterinary Medicine, Warsaw University of Life Sciences–WULS, Nowoursynowska 159, 02-776 Warsaw, Poland; 2 Witold Stefański Institute of Parasitology, Polish Academy of Sciences, Twarda 51/55, 00-818, Warsaw, Poland; 3 Department of Pathology and Veterinary Diagnostics, Institute of Veterinary Medicine, Warsaw University of Life Sciences-WULS, Nowoursynowska 159, Warsaw, 02-776 Poland; 4 Veterinary Diagnostics Laboratory ALAB plus – ALAB bioscience, Stępińska 22/30, 00-739 Warsaw, Poland; 5 Department of Animal Genetics and Conservation, Institute of Animal Sciences, Warsaw University of Life Sciences - WULS, Ciszewskiego 8, 02-787 Warsaw, Poland

**Keywords:** IKC outbreak, Bison bonasus, Ocular lesions, Eyeworm

## Abstract

**Background:**

Infectious keratoconjunctivitis (IKC) is a common ocular disease of ruminants worldwide. Recently, an outbreak of infectious keratoconjunctivitis was observed in the European bison in Poland. Hundreds of animals show conjunctival congestion, corneal opacity, and ulceration, leading to total blindness. The present study aimed to examine the ocular changes of European bison and patterns of the disease occurrence with special emphasis on the role of *Thelazia* nematodes in the development of IKC.

**Results:**

The eyes of 131 European bison, showing ocular signs and clinically healthy, were collected in Poland in 2021 – 2022 and subjected to parasitological and histopathological examination. Histopathology showed varying lesions, including corneal erosions or ulcers, diffuse purulent infiltrates to lymphocytic infiltration in the cornea, and lymphocytic or mixed conjunctivitis with CALT stimulation. The severity of ocular changes was higher in European bison from mountain areas and during the winter season. Two species of *Thelazia* nematodes – *T. skrjabini* and *T. gulosa* have been isolated from eyes. Prevalence of infection reached over 66.4%, and the infection intensity ranged from 1 to 16 nematodes per individual. Although nematodes of the genus *Thelazia* were prevalent in European bison, their occurrence did not correspond with the severity of ocular changes.

**Conclusions:**

The results of our studies allowed to identify patterns related to the first outbreak of infectious keratoconjunctivitis in European bison. Living in mountain areas and winter season were the most predisposing factors for the development of ocular changes. Despite the high prevalence of *Thelazia* nematodes in the present study, their role in forming ocular lesions was not confirmed.

**Supplementary Information:**

The online version contains supplementary material available at 10.1186/s12917-024-04375-3.

## Background

Infectious keratoconjunctivitis (IKC) is a polietiological ocular disease affecting ruminants worldwide. The disease is characterized by mild conjunctivitis with discrete lacrymation to severe keratitis with corneal perforation, resulting in irreversible blindness, associated behavioral changes, and eventually death [1–4]. The disease results in high mortality in wild ruminants, up to 30% [5]. IKC has been known for nearly a century and has been reported to occur in wildlife in a few European countries, including Switzerland, Italy, France, Austria, Slovenia [6], Spain [7] and Norway [8]. Multiple infectious agents are known to be involved in the IKC development, depending on the ruminant species [5]. *Mycoplasma conjunctivae* and bacteria from the genus *Chlamydia* were responsible for IKC in sheep and wild and domestic caprinae in Europe, North America, and India [3, 9–13]. In addition, several other bacterial agents such as *Moraxella* spp., *Klebsiella* spp., *Listeria* spp., *Staphylococcus* spp., and *Neisseria* spp. have also been isolated from animals with IKC [1, 4, 6, 14]. Viral agents, including alphaherpesviruses, were associated with ocular lesions in semi-domesticated reindeer in Norway [15]. Nematodes of the genus *Thelazia* might also be responsible for ocular lesions [16]. However, the role of parasites in the IKC development has usually been underrated [17].

Nematodes of the genus *Thelazia* are common ocular parasites located in the conjunctival sac, tear ducts, under the nictitating membrane, and on the surface of the cornea of wild and domestic animals worldwide [18, 19]. Flies of the genus *Musca* are considered intermediate hosts of *Thelazia* nematodes*.* Parasite larvae are ingested by insects with ocular and nasal discharge and develop into an infective stage. Flies pass larvae on the eyes of the definitive host during feeding [16]. Most reports of *Thelazia* nematodes in Europe refer to *T. callipeda*, associated with carnivorous animals and known for its zoonotic importance. [20–22]. Species typical for ruminants – *T. gulosa*, *T. skrjabini*, and T. rhodesi have been commonly isolated from livestock in Europe in the XXth century [19, 23–25]; however, currently, there is little data about the epidemiology of this parasite and its spread among domestic and wild herbivores [26]. Little data is available about ocular lesions forming due to *Thelazia* infection, as well as the possible role of parasites in the development of IKC.

Although European bison (*Bison bonasus*) became extinct in the wild at the beginning of the XXth century, the species was successfully restored and reintroduced to several European countries [27]. Poland is inhabited by 2600 European bison, which constitute almost 25% of its worldwide population and remain the center of European bison reproduction [28]. The recent dynamic increase of the European bison population resulted in its classification as a near-threatened species instead of an endangered one (IUCN, 2022). However, the increasing density of animals and their migration and climate change favor the transmission of pathogens between European bison and other ruminant species, resulting in new diseases and threats [29].

Recently, numerous cases of IKC have been observed in European bison in Poland, especially in the Bieszczady Mountains. Animals show anatomopathological changes in eyes, including conjunctivitis, conjunctival congestion, and corneal opacity and ulceration, resulting in vision impairments leading to total blindness [30]. This study aims to provide a histopathological description of the observed ocular changes formed in the different stages of IKC in European bison and to determine patterns of IKC occurrence with special emphasis on *Thelazia* nematodes as one of the potential causes of the disease.

## Results

### Study population

The study population comprised 131 European bison, 77 males and 54 females, aged from 5 months to 22 years (median 13 years). The median age of the males and females was 13 and 11.5 years, respectively. 87 individuals from the Bieszczady Mountains belonged to the Lowland-Caucasian line, whereas 44 animals from north-eastern Poland represented the Lowland line. For 11 animals, only one eye was possible to collect.

European bison from the Bieszczady Mountains were eliminated due to visible changes in eyes, whereas animals from north-eastern Poland were culled mainly due to injuries, traumas, and other diseases.

### Infection of *Thelazia* nematodes

Nematodes of the genus *Thelazia* were isolated from 87 of 131 European bison, i.e. the prevalence reached 66.4%. Unilateral infection was observed in 47 animals, whereas 30 European bison showed the presence of *Thelazia* nematodes in both eyes. In other infected European bison, only one eye was examined. The most prevalent species was *T. skrjabini*, detected in 61.1.% of European bison, whereas *T. gulosa* was isolated only from 15.5% of individuals (Table 1).

**Table 1:** Prevalence and intensity of infection of *Thelazia* nematodes in eyes of European bison

Parasite	Overall			Right eye		Left eye	
	Number of infected bison	Prevalence (CI 95%)	Median intensity of infection (range)	Number of infected bison	Median intensity of infection (range)	Number of infected bison	Median intensity of infection (range)
*Thelazia* spp.	87/131	66.4% (62.1–70.7)	2 (1–16)	63/124	2 (1–12)	60/127	2 (1–6)
*T. skrjabini*	80/131	61.1% (52.8–69.4)	2 (1–12)	55/124	2 (1–9)	54/127	2 (1–5)
*T. gulosa*	20/131	15.3% (9.1–21.5)	2 (1–10)	16/124	1.5 (1–10)	11/127	2 (1–6)

Co-infections with *T. gulosa* and *T. skrjabini* were observed in 13 animals (9.9%). If co-infection occurred, there was a higher chance for bilateral *Thelazia* infection (Fisher's Exact Test for Count Data *p* < 0.001). There were no significant differences between the prevalence of *Thelazia* species and age, sex, or genetic line of examined European bison, as well as the season of sample collection and presence or severity of ocular changes (Chi square = 4.45, *p* = 0.348).

**Fig. 1:**
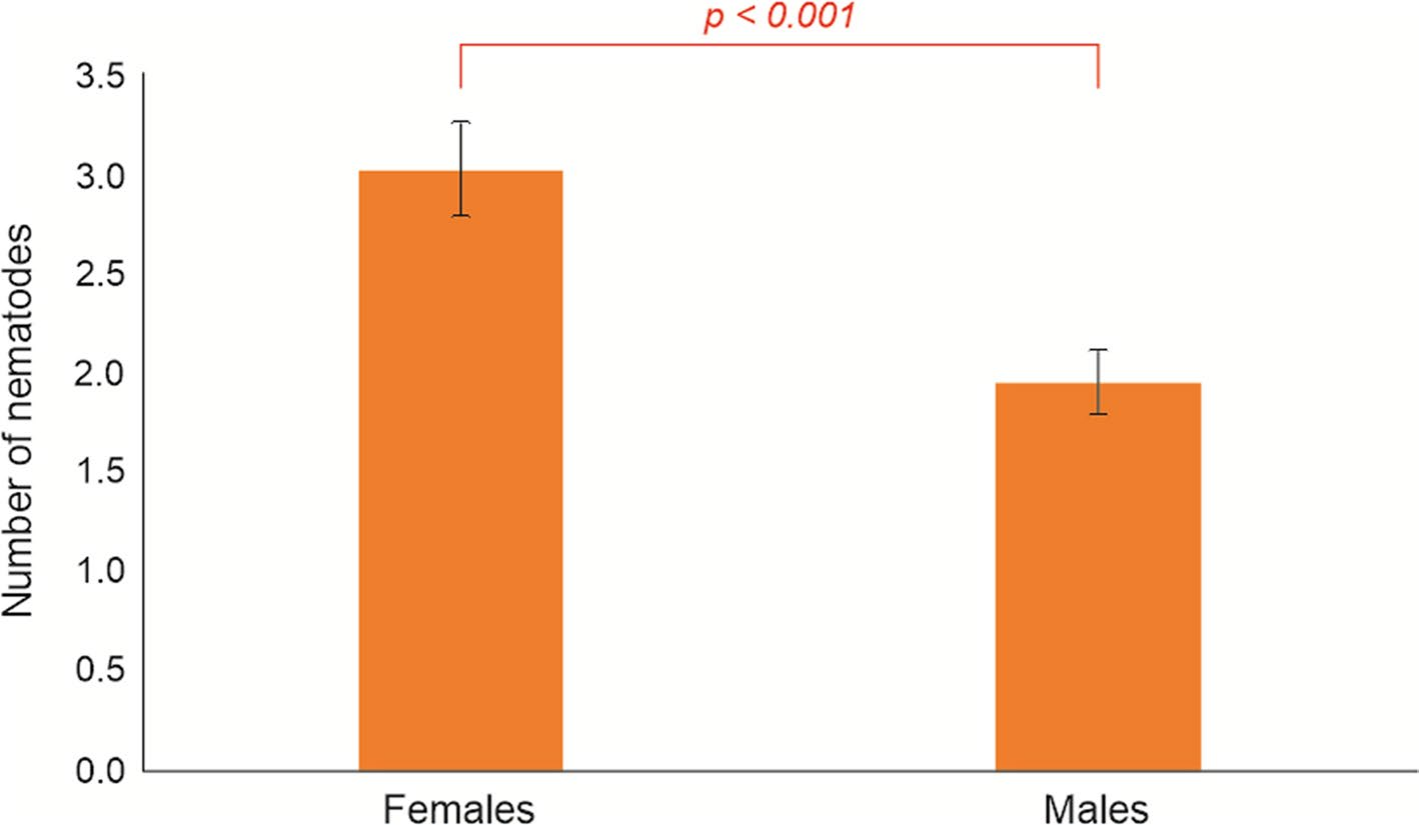
Intensity of infection of *Thelazia* nematodes in female and male European bison

The intensity of infection ranged from 1 to 16 and differed significantly between animals infected with single *Thelazia* species and those with co-infections (U-Mann Whitney test = 129.5, *p* < 0.001). The number of isolated nematodes did not differ significantly between the left and right eye (Wilcoxon Z test = −0.75, *p* = 0.453, Table 1). At the same time, infection intensity with *Thelazia* spp. in the left eye was positively correlated with the right one (*r* = 0,365, *p* < 0.001), which was also observed for *T. skrjabini* (*r* = 0,285, *p* = 0.002), but not for and *T.gulosa* (*r* = 0.168, *p* = 0.067). There were no significant differences between the intensity of *Thelazia* nematodes in relation to age and genetic line of European bison, as well as the season of sample collection and the presence or severity of macroscopic and histopathological ocular changes. The intensity of infection with *Thelazia* nematodes was significantly higher in females than males (Chi-square = 14.94, *p* < 0.001 for the whole model) (Fig. 1).

### Molecular examination of *Thelazia* nematodes

Species of *T. skrjabini* was confirmed by amplification of 640 bp fragment of the mitochondrial cytochrome c oxidase subunit 1 gene (cox1). Obtained sequences were submitted to the GenBank database (GenBank accession no: PP835152; PP835153). BLAST analysis revealed their identity with sequences obtained from European bison in Poland during previous studies (GenBank accession no: OL362009.1) as well as isolates of *T. skrjabini* from farmed American bison (*Bison bison*) in Romania (GenBank accession no: OR673496.1). Over 99% similarity with sequences of *T. skrjabini* obtained from cattle in Romania has also been observed (GenBank accession no: OQ988149.1, OQ988150.1, OQ988151.1, OQ988154.1, OQ988156.1).

Amplification of *T. gulosa* was not successful.

### Anatomopathological analysis

**Fig. 2:**
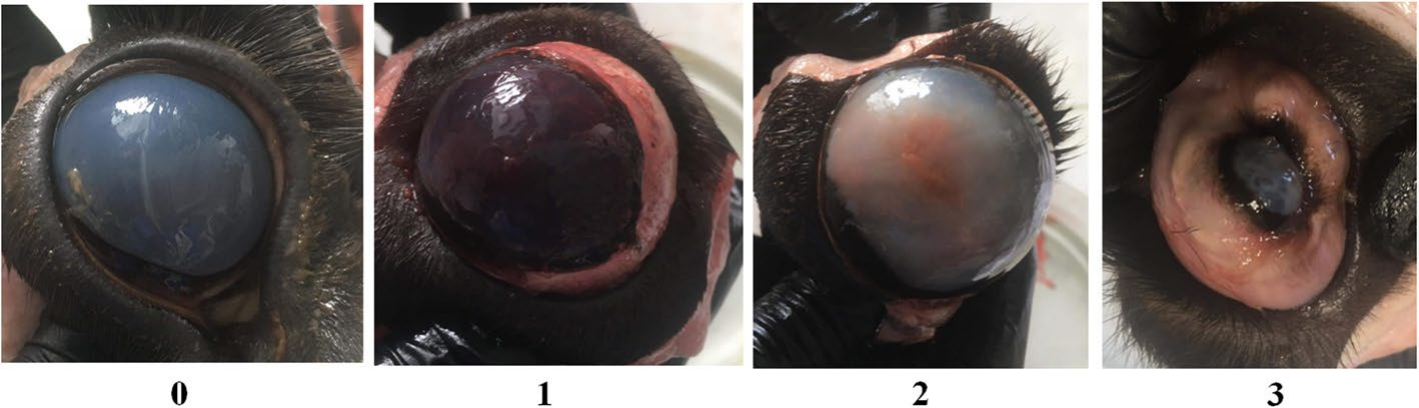
The severity of macroscopic ocular changes observed in European bison scored from 0 to 3, with 0 for healthy eyes, 1 for increased lacrimation and/or mild conjunctivitis, 2 for animals with moderate to severe clinical signs of IKC and 3 for severe changes (deformation of the eyeball)

Gross lesions of different severity (Fig. 2) were observed in at least one eye of 121 among 131 European bison, i.e., 93.4% of examined animals. 74% of animals showed advanced changes, including corneal opacity, ulceration, and purulent eyeball inflammation, whereas extremely severe lesions, like eyeball deformation, were observed in 16.8% of European bison. Hyperemia and conjunctivitis were the least often. The severity of macroscopic ocular changes was positively correlated between the left and right eye (Pearson’s r = 0.49, *p* = 0.001).

**Fig. 3:**
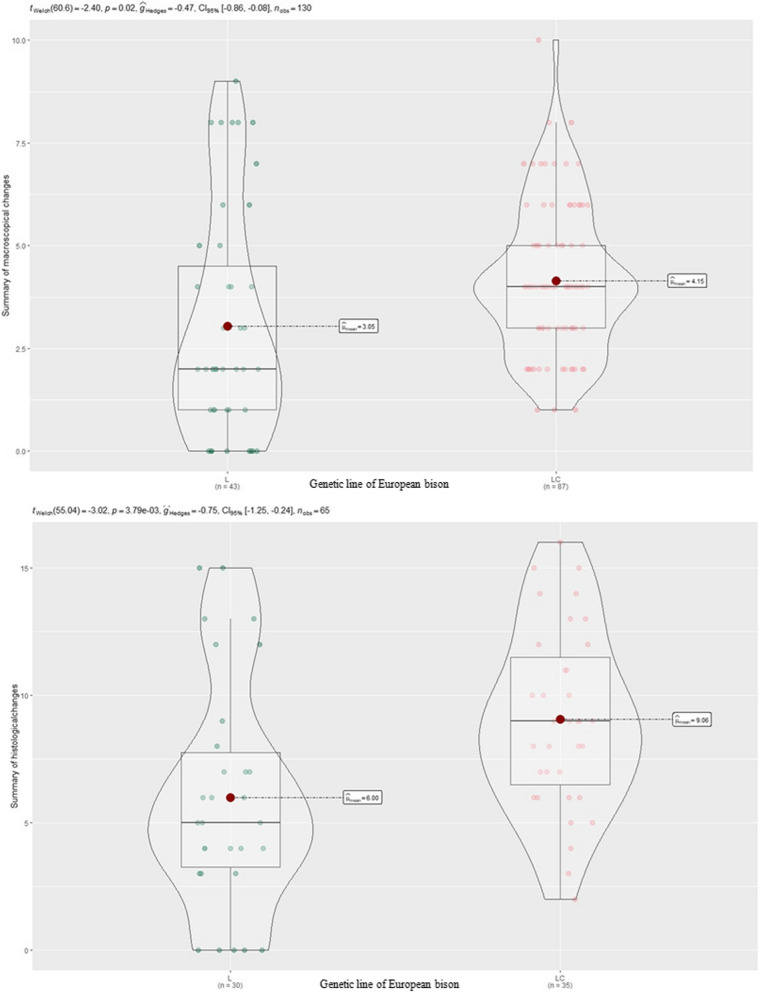
Severity of macroscopical and histopathological changes in European bison of the Lowland and the Lowland-Caucasian line

There were no statistically significant differences between the occurrence and severity of ocular changes and age, sex, or weight of animals; however, more severe lesions were observed in the eyes of European bison from the Bieszczady Mountains, thus belonging to the Lowland-Caucasian line in comparison with animals of the Lowland line (*p* = 0.001) (Fig. 3).

### Histopathology

**Fig.4:**
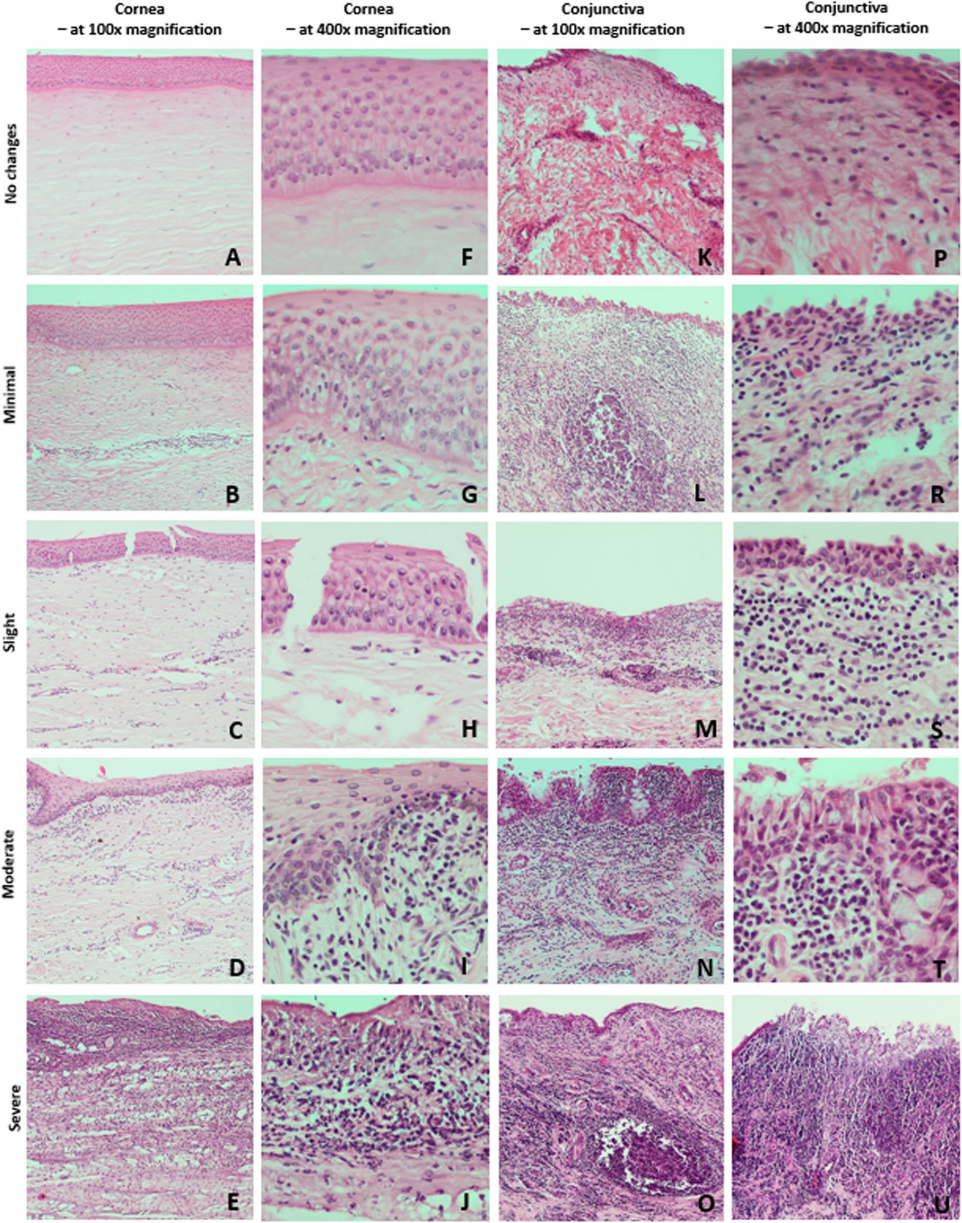
Intensity of histopathological changes in IKC in the European bison. Presentation of the cascade of severity of pathological changes in the cornea and conjunctiva according to the applied scalar severity assessment system. Legend of the scalar assessment of the severity of histopathological changes for the cornea: No changes—sum of points: 0–5; Minimal—sum of points:6–12; Slight—sum of points: 13–19; Moderate—sum of points: 20–26; Severe—sum of points: more than 27; for conjunctiva: No changes—sum of points: 0–10; Minimal—sum of points: 11–20; Slight—sum of points: 21–30; Moderate—sum of points: 31–40; Severe—sum of points: more than 41

The severity of histopathological changes in the cornea and conjunctiva are presented in Fig. 4.

**Fig.5:**
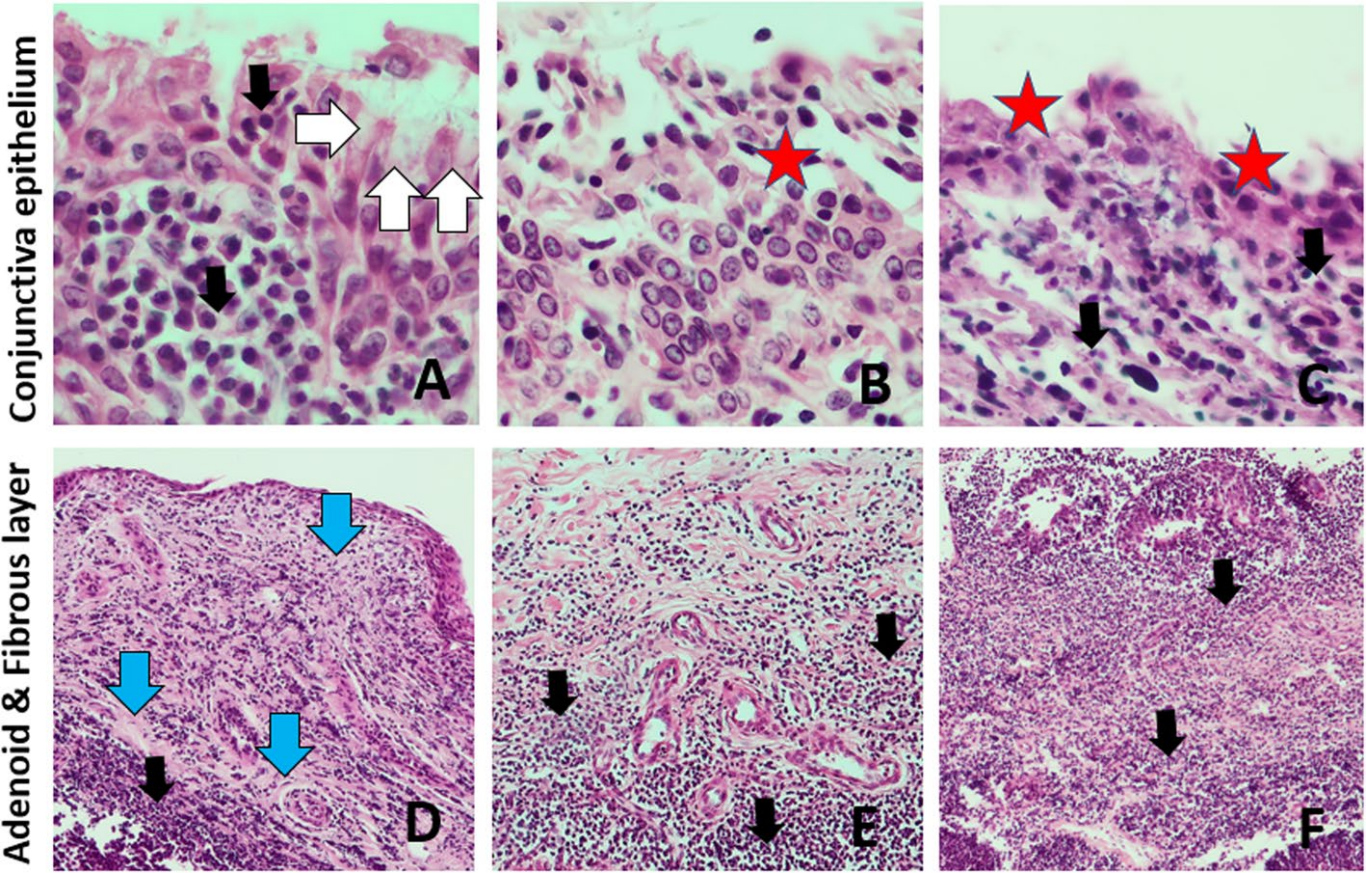
Types of histopathological changes observed in the conjunctiva in IKC in the European bison. A-C – conjunctival epithelium in magnification × 400; D-F – conjunctival stroma in magnification × 100. Black arrow—mononuclear cell infiltration, White arrow—hyperplastic goblet cells, Blue arrow—fibrosis of the conjunctival stroma and the formation of connective tissue bundles, Red star – exfoliation and delamination of the conjunctival epithelium, Yellow star—stimulated lymphatic follicles

In the conjunctiva, erosions, and ulcerations of the epithelium, especially in areas of inflammatory infiltrates, as well as epithelial cells shedding together with less frequent hyperplasia of epithelium and goblet cells, were observed. Infiltration of lymphocytes with a component of plasma cells and neutrophils in the submucosa and mucosa propria, as well as perivascular inflammatory infiltrates, including periarterial and lacrimal glands were also found. Swelling of the mucosa propria, followed by subepithelial connective tissue hyperplasia or fibrosis and hyperplasia of CALT lymph nodes, were observed in more severe cases (Fig. 5).

**Fig. 6:**
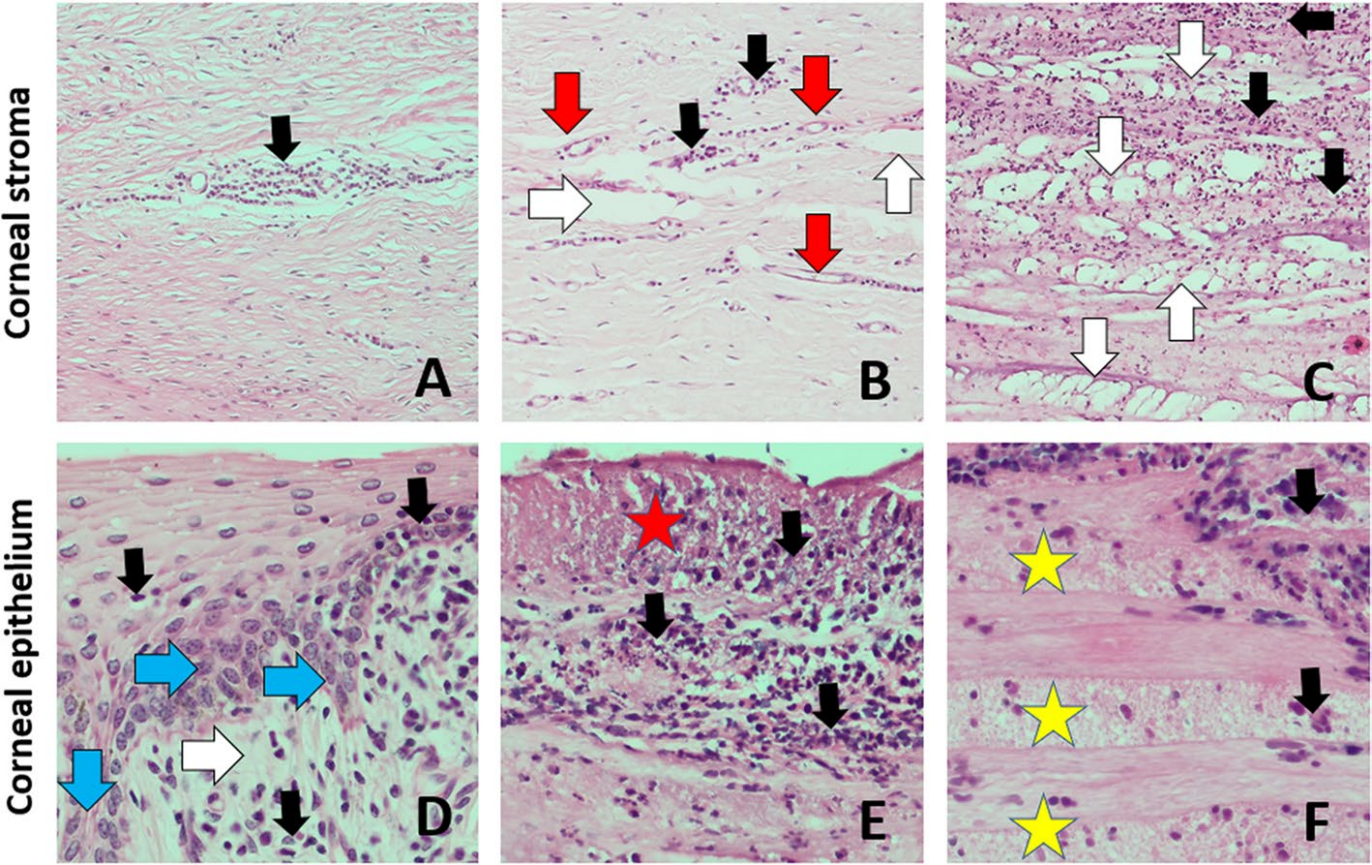
Types of histopathological changes observed in the cornea in IKC in the European bison. A-C – corneal stroma in magnification × 100; D-F – corneal epithelium in magnification × 400. Black arrow—mononuclear cell infiltration, Red arrow—neovascularization, White arrow—edema and delamination of corneal stroma fibers, Blue arrow—epidermal metaplasia of the corneal epithelium, Red star – necrosis of corneal epithelium, Yellow star—layered necrosis of the subepithelial corneal stroma fibers

The most common histopathological changes of the cornea were erosions and exfoliations of anterior corneal epithelial cells followed by less frequent cell vacuolation and swelling as well as intraepithelial and subepithelial cysts and epithelial delaminations. Focal, circumscribed full-thickness ulcers of the anterior corneal epithelium were observed less frequently, mostly in severely changed eyeballs. In the substantia propria of the cornea, swelling of fibers followed by disorder of fibers architecture with delaminations and cavities in the substantia propria were also commonly observed. Neovasculogenesis and fibroplasia were found in more severe cases (Fig. 6). Thickening of Descemet's and Bowman’s membranes was also found commonly. Infiltration of lymphocytes, less frequently neutrophils and histiocytes, in the anterior corneal epithelium and the substantia propria of the cornea were observed less frequently.

There was a significant correlation between the severity of histopathological changes in the left and right eye (*r* = 0.5). More severe histopathological lesions were observed in European bison from the Bieszczady Mountains, belonging to the Lowland-Caucasian line (Pearson’s Chi-squared test *p* = 0.003) (Fig. 3). There was no significant relationship between the severity of histopathological changes and the sex or age of animals; however European bison showed more serious gross lesions during the winter season (Pearson’s Chi-squared test *p* = 0.003).

Statistical analysis of the morphometric parameters of the cornea did not reveal significant differences between groups of animals, divided according to the severity of histopathological changes. Nevertheless, a tendency for lower corneal epithelium in severely changed eyeballs in comparison with eyeballs showing no pathological changes, can be observed.

Additionally, in severely changed eyeballs (score 4), Bowman's membrane thins or even atrophies, unlike Descemet's membrane, which thickens. In animals with moderate or severe ocular lesions, the thickness of the substantia propria increases (Additional file 1).

## Discussion

The primary aim of this study was to describe and identify patterns associated with the occurrence of IKC in European bison from southern and northeastern Poland. During the present study, over 93% of examined animals showed visible ocular lesions in at least one eye. Detection of early stages of the disease in wildlife is difficult. Thus, animals with easily visible ocular lesions were predominantly eliminated and examined during the present study. 74% of European bison showed advanced stages of the disease in at least one eye, whereas 16.8% of animals suffered from extremely severe changes, including eyeball deformation. According to Giacometti et al. (2002) [6], severe signs of IKC occur more frequently in wildlife, which can be the cause of significant mortality in wild ruminant populations [31]. In contrast, domestic ruminants with IKC are often asymptomatic or mildly symptomatic [32]. Changes in the eyes of European bison were consistent with descriptions of IKC provided by other authors, including congestion and fibrous deposits in the conjunctiva as well as epithelial hyperplasia, lymphocytic-histiocytic inflammatory infiltration, neovascularization, and ulceration of the cornea [4, 33, 34]. Lesions were limited to the eye's anterior chamber except in most severe cases with deformed eyeballs. Moreover, histopathological findings were consistent with clinical signs and severity of macroscopic lesions. There was a positive correlation between the occurrence and severity of ocular lesions in the left and right eye, possibly due to easy disease transmission to the adjacent tissues through secretions and by flying vectors [15, 35]. Therefore, IKC in one eye might be quickly transmitted and predispose the animal to bilateral disease, which is the most common in ruminants [32]. It is consistent with our studies as the majority, i.e., over 81% of examined European bison, showed bilateral ocular lesions.

Generally, more severe lesions were observed in European bison of the Lowland-Caucasian line, inhabiting the Bieszczady Mountains in southern Poland. Ocular changes were the main reason for eliminating animals from this area. It might result from high animal density in the Bieszczady Mountains, exceeding the carrying capacity of the studied area [36]. It favors disease transmission through direct contact between animals and eye-frequenting flies.

Additionally, European bison is considered an extremely inbred species [37], making it more vulnerable to infectious diseases. Lower resistance to IKC has already been observed in some breeds of cattle [38] and might also be one of the reasons for common disease occurrence in European bison in the Bieszczady Mountains. According to Tokarska et al. (2009) [39], signs of inbreeding depression are evident in the Lowland-Caucasian line of European bison; however, the Lowland line also shows a high inbreeding coefficient.

According to other authors, altitude might negatively affect the course of IKC [13] due to higher ultraviolet radiation [40] and air dryness in high mountain ranges [41]. Therefore, outbreaks of IKC in Europe often occurred in mountain habitats [5, 6, 13], which is consistent with our results.

Several other predisposing environmental factors favor the spread of IKC, depending on the pathogen involved in the disease development [33, 34, 42]. In our studies, animals tended to have more advanced histopathological changes in eyeballs during the winter season. Other authors reported clinical IKC cases mostly during autumn and winter [4, 42, 43]. It might be connected with increased animal movements and aggregation of animals in winter or higher stress levels and immunological deficiencies due to harsh environmental conditions. Snow cover probably additionally increases the negative impact of ultraviolet radiation [40] on the development of ocular changes. IKC outbreaks were also observed by other authors during summer, possibly as a cause of the increased activity of eye-frequenting flies, which favor the mechanical spread of ocular pathogens [35, 44, 45]. According to Philippot et al. (2023) [42], summer-biased IKC is more common in lowland wild ruminants inhabiting forests, not mountain areas. However, it was not observed in our study. On the other hand, higher precipitation and annual temperature in the Bieszczady Mountains, in comparison with northeastern Poland, might favor the survival of eye-frequenting flies and transmission of pathogens connected with IKC.

Nematodes of the genus *Thelazia* are considered one of the possible causes of keratoconjunctivitis [16]. In the present study, parasites were isolated from the eyes of over 66% of European bison. It is similar to previous studies of European bison in Poland [19, 30]; however, little data about thelasiosis in wildlife is generally available. The prevalence of eyeworms in European bison was significantly higher than in domestic ruminants in other parts of Europe [26]. Wild herbivores might be especially susceptible to *Thelazia* infection as they are highly exposed to the eye-frequenting flies, which transmit the parasite [16].

In the present study, two nematode species were isolated from European bison. Most prevalent was *T. skrjabini*, followed by less frequent *T. gulosa*, both typical for ruminants and isolated from European bison before [19, 30].

Although no sequences of *T. gulosa* have been obtained during the present study, the morphological identification of the species was performed according to Demiaszkiewicz et al. (2020) [19] and should be considered reliable. Sequences of *T. skrjabini* were similar to isolates of nematodes from American bison (GenBank accession no: OR673496.1) and cattle (GenBank accession no: OQ988149.1, OQ988150.1, OQ988151.1, OQ988154.1, OQ988156.1) in Romania, which might be evidence of interspecies transmission of *Thelazia* nematodes between different wild and domestic ruminants.

Co-infections with both *T. skrjabini* and *T. gulosa* were observed in 13 animals; however, there were no statistically significant differences between the intensity of infection and the severity of ocular lesions in European bison, whether infected with one or both species. Nevertheless, thelasiosis more likely covers both eyes of European bison if co-infection occurs. It might result from high exposure of European bison to pathogenic nematode due to the sympatric occurrence of both species in the environment [41], which could possibly increase the risk of secondary bacterial infections and the development of pathological changes.

In southern Europe, *T. rhodesi* is the most abundant species in livestock [25, 26, 46]. It has also been detected in European bison in Romania (GenBank accession no: OR673501.1). However, there is no current data about its occurrence in wild or domestic ruminants in Poland.

Despite the high prevalence, the overall median intensity of *Thelazia* infection in European bison did not exceed two nematodes and was slightly lower compared with other studies [25, 26]. Females were infected with a higher number of nematodes than bison bulls. Female-biased parasitism has been well described in populations of European bison, as the concentration of females living in herds possibly favors the transmission of parasites [27, 47, 48]. A similar tendency has also been observed during previous studies [30].

Statistical analysis revealed that the infection intensity of *Thelazia* nematodes in the right eye was significantly correlated with infection in the left eye, which indicates that the parasite might be easily transferred. A similar tendency was observed for Cervid Herpesvirus 2 in the infected semi-domesticated reindeer [15].

Despite the seasonal tendency for higher infection intensity of *Thelazia* nematodes in summer, reported in domestic ruminants by other authors [16, 49], it was not observed in our study. It might result from severe ocular changes in European bison, which limited the nematode’s survival and disturbed the impact of flying vectors and other environmental factors on its seasonal occurrence and transmission. Additionally, the exposition of European bison as wild ruminant species on the flying vectors might be higher than that of livestock subjected to farm management and seasonal antihelmintic treatment. Therefore, some tendencies for *Thelazia* occurrence in European bison might differ from the available data. No relationship between the prevalence and intensity of *Thelazia* infection and the occurrence and severity of ocular changes was observed in European bison in the present study. The presence of nematodes did not differ between studied locations despite significantly higher severity of the observed ocular changes in the Bieszczady Mountains. As IKC is a polietiological disease, thelaziosis could represent a predisposing factor in its pathogenesis, exacerbating the effect of bacterial and viral ones [5, 32, 43]. As most samples were collected from animals with advanced IKC, revealing the primary and secondary infectious agents causing the disease might be difficult. According to Otranto and Traversa (2005) [16], *Thelazia* nematodes might be one of the potential factors involved in the development of bovine keratitis. Therefore, the common occurrence of eyeworms in the eyes of European bison should not be neglected, as their partial role in the initiation or progress of IKC is possible.

On the other hand, no data about the current spread of *Thelazia* nematodes in clinically healthy European bison is available, despite their occurrence in the 50 s and 80 s [50, 51]. As animals with ocular changes dominated in the present study, it is hard to conclude the role of nematodes in the development of IKC without examination of European bison, which do not show signs of the disease.

## Conclusion

Our study describes histopathological changes formed in the eyes of European bison in the course of IKC, together with an attempt to identify patterns associated with the occurrence of the disease in an endangered ruminant species. European bison from the Bieszczady Mountains, southern Poland, seem to be especially exposed to the development of ocular changes, possibly due to the negative impact of altitude and ultraviolet radiation; however, the role of other factors should not be neglected. Therefore, the Bieszczady Mountains should be considered as the main area of the IKC outbreak. Despite the high prevalence of *Thelazia* nematodes, further studies are needed to define the role of parasites in the development of ocular changes. Nevertheless, eyeworms might be more common in the population of European bison than previously expected. It is necessary to perform further studies to understand the epidemiology of IKC in European bison and identify infectious agents associated with the occurrence and progression of the disease. The influence of populational and individual factors, such as the high density of herds and the genetic predisposition of animals, should also be investigated. Examination of clinically healthy European bison and other ruminant species in the studied area and examining flying vectors would help identify patterns of IKC transmission and thus exposure of the wild ruminant population to this dangerous disease.

## Materials and methods

### Study area

The primary location of the material collection is the Bieszczady Mountains in southeastern Poland. The landscape of the Bieszczady Mountains is characterized by long mountain ranges running from the north-west to the south-east. The climate is temperate with dominants of continental features. The mean annual temperature reaches 7.5℃. The precipitation ranges from 800 to 1200 mm. The mean high above the sea level is 1 405 m. The Polish part of the Bieszczady Mountains is inhabited by 750 European bison of the lowland-caucasian line (LC), which constitutes the second biggest free-living population of this mammal in Poland.

The four other study areas, namely the Białowieża Primeval Forest, the Knyszyn Forest (53°10′35″ N, 23°48′47″ E), the Borecka Forest and the Augustów Forest (53°58′46″ N, 23°17′23″ E) are located in northeastern Poland with transitional temperate climate zone, dominated by continental influences. The mean annual air temperature ranges from 6 to 7 °C. Precipitation varies around 550—650 mm, and mean altitude does not exceed 220 m n.p.m. Białowieża Primeval Forest is inhabited by about 830 European bison, Poland's biggest population. 300, 120, and 20 European bison are located in the Knyszyn, Borecka, and Augustów Forests. All European bison in north-eastern Poland belong to the Lowland line (L).

### Material collection and parasitological examination of eyes

In the years 2021–2022, 131 European bison from the Bieszczady Mountains (87 individuals), Białowieża Primeval Forest (31 individuals), Knyszyn Forest (9 individuals), Borecka Forest (3 individuals) and Augustowska Forest (one individual) were eliminated due to vision impairments and visible changes in eyes as well as traumas and other injuries.

All European bison were legally culled based on permission from the General Directorate for Environmental Protection in Poland. The decision of the General Directorate for Environmental Protection was implemented by the Regional Head Offices of National Forests, which supervised animal sacrifice. As wild animals, European bison were culled by professional hunters with respect to the animal's welfare and safety rules in the presence and assistance of qualified veterinarians.

Animals were divided into two groups depending on the location and thus genetic line: the lowland-caucasian line for European bison in the Bieszczady Mountains and the lowland line for animals from forests of north-eastern Poland (Białowieża, Knyszyn, Borecka, and Augustowska Forest).

During the post-mortem examination, the age, sex, and weight of animals were estimated. Both eyeballs and adjacent tissues were collected from each individual, preserved in 70% ethanol, and transported to the laboratory at 4℃.

Eyes were then subjected to parasitological examination by rinsing the conjunctival sac, tear ducts, corneal surface, and nictitating membrane with saline solution. Nematodes of the genus *Thelazia* were isolated under the stereoscopic microscope from the decantated sediment, identified based on morphological features [19], and preserved in 70% ethanol for further molecular analysis.

### Molecular investigation of *Thelazia* nematodes

Genomic DNA was extracted from 5 worms using a Nucleospin Tissue DNA extraction kit (Macherey–Nagel, Germany). The worms' partial nucleotide sequences of the mitochondrial cox1 gene were amplified following Filip-Hutsch et al., 2022 [29]. The set of primes used involved forward-COIintF (5’-TGATTGGTGGTTTTGGTAA-3’) and reverse-COIintR (5’-ATAAGTACGAGTATCAATATC-3’). The PCR was performed in a T100 thermal cycler (Bio-Rad, USA) in a volume of 50 ul following Filip-Hutsch et al., 2022 [30]. The conditions for PCR were modified as follows: 94C for 2 min, 35 cycles at 94C for 40 s, 55C for 45 s, and 72C for 45 s; and the final extension at 72C.

The PCR products were purified with the use of the Nucleospin Gel and PCR Clean-up kit (Macherey–Nagel), eluted with 30 ul of molecular biology reagent water, and sequenced in both directions by Genomed S.A. (Warsaw, Poland). The sequences were assembled into contigs using CodonCode Aligner ver. 8.0 (CodonCode Corporation, USA).

### Anatomopathological analysis

Macroscopic evaluation of eyes was performed during the field dissections by qualified hunters and veterinarians. The severity of the ocular disease was scored from 0 to 3, with 0 for healthy eyes, 1 for increased lacrimation and/or mild conjunctivitis, 2 for animals with moderate to severe clinical signs of IKC (keratitis and conjunctivitis, corneal ulceration, purulent ocular inflammation, fibrin deposits) and 3 for severe changes (deformation of the eyeball).

### Preparation of histological slides

Histopathological assessment was performed for 124 eyes of 76 European bison. After parasitological examination, the eyeballs, together with adjacent conjunctival sacs, were subjected to an initial macroscopic evaluation to assess the degree of fixation. If they were insufficiently fixed or fixed in 70% ethanol, they were transferred to 10% buffered formalin for 72 h. After fixation, a full cross-section of the anterior pole of each eyeball and a second cross-section perpendicular to the first one were prepared. In addition, three fragments of the conjunctival sac were collected, including a fragment of the conjunctival membrane covering the medial angle and the third eyelid. Then tissue sections were macroscopically examined and dehydrated in graded ethanol and xylene baths. The dehydrated sections were then embedded in paraffin wax and were cut on a microtome. The sections (measuring 3–4 µm) were stained with haematoxylin and eosin (H-E). Microscopic evaluation was performed at 5x, 10x, and 40 × magnification, and the sample was photographed. Tissue sections were examined using an Axiolab A5 light microscope with Axiocam 208 color and ZEN 3.0 software (Zeiss, Jena, Germany).

### Histopathological examination and scalary evaluation of changes

The location, type of pathological change, and severity of ocular changes were assessed by professional veterinary pathologists. An evaluation protocol was created for each tissue (eyeball and conjunctiva) for standardization of the histopathological evaluation between the different researchers. Histopathological changes in the eyeballs were graded on a five-grade severity scale, with a point score for each major structure including anterior corneal epithelium, corneal stroma, Descemet's membrane, corneo-iris angle, the anterior chamber space of the eye, were subjected for analysis. The evaluation for the conjunctiva included conjunctival epithelium, substantia propria (glandular and fibrous layers), and conjunctiva-associated lymphoid tissue (CALT). Two scales were used according to the type of the observed changes: scale B for the severity of structural changes and scale C for the infiltration level. Each histopathological change included in the evaluation protocol was graded in terms of severity according to the scale: 0—no change, 1—minimal, 2—slight, 3—moderate, 4 – severe (Additional file 2).

On the basis of severity of pathological findings and sum score on a given scale, an overall score for a given eyeball and conjunctival sac was established with 0 being normal tissue and 4 being severe changes.

### Morphometric measurements

Morphometric measurements were performed on 112 eyeballs from 62 individuals. Due to severe anatomopathological changes, it was not possible to identify anatomical structures suitable for quantitative morphological measurements in other collected eyeballs. Morphometric measurements were performed using an Axiolab A5 optical microscope, an Axiocam microscope camera, and a ZEN Zeiss histological image analysis program. The following parameters were analyzed: height of anterior corneal epithelium, Bowman's membrane, substantia propria, Descemet's membrane and number of cells in the anterior epithelium.

### Statistical analysis

*Thelazia* infection was analyzed with SPSS software (PS Imago Pro 10.0). Fisher’s Exact Test was used for the connection of bilateral infection with the presence of both *Thelazia* species. We used the U-Mann–Whitney test to compare the intensity of infection with single *Thelazia* species and animals with co-infection. Wilcoxon Z test compared the number of isolated nematodes between the left and right eye. We also used Pearson's correlation coefficient to assess the correlation of nematode numbers in the left and right eye. We performed three such correlations, separately for two nematode species: *T. skrjabini* and for *T.gulosa* and for both species together. We applied a generalized linear binary model for the assessment of the prevalence of *Thelazia* spp., where the presence (marked as 1) or absence (marked as 0) of nematodes was the dependent variable, and age, sex, genetic line, season, and severity of ocular changes were the explanatory variables. We applied a generalized linear model with Poisson distribution and log link function to assess the intensity of infection of *Thelazia* spp., where the number of nematodes was the dependent variable, and age, sex, genetic line, season, and severity of ocular changes were the explanatory variables. We used full model selection to find the best-fit model using the Akaike Information Criterion (AIC).

Anatomopathological changes were analyzed in R version 4.4.0 [52]. Outliers in the dataset were detected by Rosner's Test for Outliers and excluded for further analyses. Categorical variables were presented as counts and percentages and compared between groups using Pearson's Chi-squared test with Yates' continuity correction, Fisher's Exact Test for Count Data, or Mantel–Haenszel's chi-squared test without continuity correction, depending on fulfilling test assumptions. For quantitative variables, a linear correlation was calculated using the Pearson correlation coefficient. Before performing tests of group comparisons, assumptions of normality, homogeneity of variances, and lack of autocorrelation were checked. Depending on the results, a proper group comparison test was performed.

The confidence interval (95% CI) for proportions was assessed using the Wilson score method. The significance level was set at 0.05.

## Supplementary Information

Additional file 1. Morphometric measurements of corneal layers. Morphometric measurements of the height of anterior corneal epithelium, Bowman's membrane, substantia propria, Descemet's membrane, and number of cells in the anterior epithelium and comparison of measurements between groups of animals showing different severity of changes.

Additional file 2. Evaluation protocol of histopathological changes of the cornea and conjunctiva. Evaluation protocol of histopathological changes of the cornea and conjunctiva.

## Data Availability

Data is provided within the manuscript or supplementary information files. Other datasets used and/or analyzed during the current study are available from the corresponding author on reasonable request.
